# Research progress on nutritional support in the neonatal and pediatric populations receiving extracorporeal membrane oxygenation

**DOI:** 10.3389/fnut.2024.1370286

**Published:** 2024-06-03

**Authors:** Hongquan Zhang, Lizhuo Zhao, Baohui Jia

**Affiliations:** ^1^Zhengzhou Railway Vocational and Technical College, Zhengzhou, Henan Province, China; ^2^Department of Emergency and ICU, Fourth Affiliated Hospital of Nanchang University, Nanchang, Jiangxi Province, China; ^3^Department of Pediatric ICU, Henan Provincial People’s Hospital, Zhengzhou, Henan Province, China

**Keywords:** extracorporeal membrane oxygen, children, nutritional support, enteral nutrition, parenteral nutrition

## Abstract

Nutritional support is crucial for the prognosis of children supported by extracorporeal membrane oxygenation (ECMO). This article discusses the latest research progress and guideline recommendations for nutritional support during ECMO. We summarize the nutritional status and evaluation of ECMO patients, nutritional support methods and timing, trace elements, the impact of continuous renal replacement therapy (CRRT), and energy requirements and algorithms. The article shows that malnutrition is high in ECMO patients compared to other critically ill patients, with nearly one-third of patients experiencing a decrease in nutritional indicators. The timing of the initiation of nutrition is very important for the nutritional status of the child. Early enteral nutrition can improve patient prognosis, which is the most commonly used, with parenteral nutrition as a supplement. However, the proportion of enteral nutrition is relatively low, and a stepwise nutrition algorithm can determine when to initiate early enteral nutrition and parenteral nutrition. Malnourishment during critical illness have been associated with increased morbidity as well as increased mortality. Nutritional status should be evaluated at admission by screening tools. In addition, changes in the levels of several metabolites *in vivo*, such as blood lipids, carnitine, and thiamine, can also reflect the degree of nutritional deficiency in critically ill children. This article provides a reference for the implementation of nutrition of pediatric ECMO patients and further research on nutritional support.

## 1 Introduction

Extracorporeal membrane oxygenation (ECMO) is an advanced technology that is used to temporarily replace cardiac or pulmonary functions and buy time for the treatment of the underlying disease. The nutritional status of pediatric ECMO patients has a significant impact on prognosis. Proper nutritional support can influence treatment outcomes and prognosis of these children. Critically ill infants and children undergoing ECMO treatment are nutritionally vulnerable, and providing adequate nutrition is essential because it has a beneficial impact on outcomes. There is no clear consensus about the optimal approach to nutritional prescribing for these patients (1). We conducted a systematic review to mainly determine whether EN and PN is effective and its association with rates of complications and mortality in critically ill neonatal and pediatric populations supported by ECMO. In short, this article provides the existing evidence of the research progress on the nutritional status of pediatric ECMO patients during treatment, methods and timing of nutrition, complications, calculation of nutrition, as well as the indicators for nutritional metabolism. These findings can provide valuable insights for the nutritional management of pediatric ECMO patients.

## 2 Materials and methods

**Figure 1:**
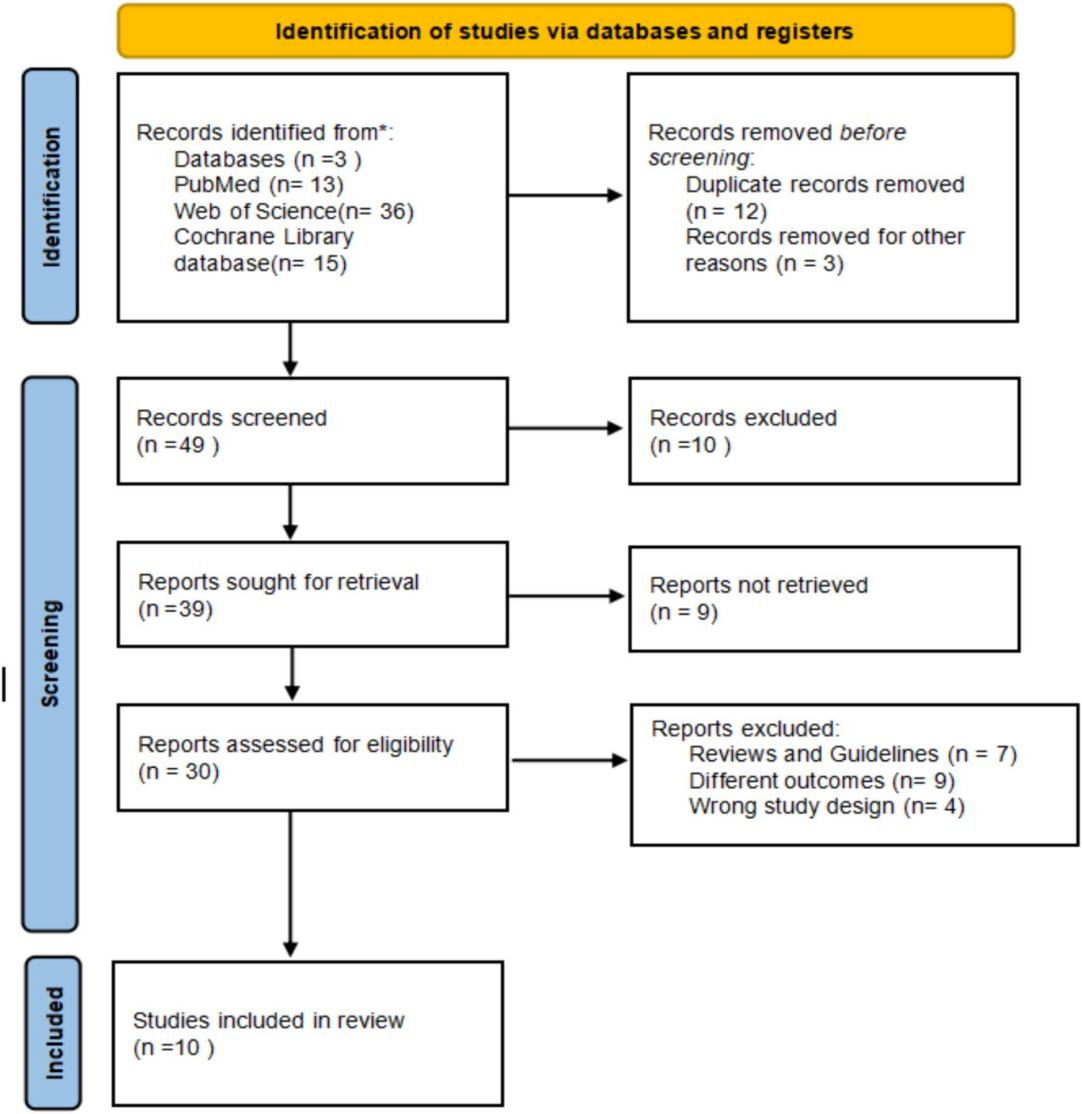
Preferred reporting items for systematic review and meta-analysis (PRISMA) flow diagram for review.

The literature review was organized according to the Preferred Reporting Items for Systematic Review and Meta-Analysis 2020 guidelines (2) (Figure 1).

### 2.1 Study selection

Studies that evaluated EN and PN in neonates and pediatric patients on ECMO were conducted. The studies also had to report either complications or mortality related to EN and PN.

### 2.2 Data sources and search strategies

The databases used in this literature review were MEDLINE, Web of Science (WOS), and the Cochrane Library database with the search terms ([Extracorporeal membrane oxygenation or ECMO or Extracorporeal Life Support or ECLS] AND [enteral nutrition or nasal feed* or tube feed* or gastric tube feed*] AND [parenteral nutrition] AND [pediatric or pediatric or child* or infant* or neonatal or newborn]) from inception to April 2024, with English language restrictions. The reference lists of reports that were potentially relevant were also searched to identify additional studies. After applying the exclusion criteria, there were a total of 10 studies that met the inclusion criteria (Table 1).

**Table 1:** Clinical characteristics, Nutrition management and results.

Studies	Study design; age	Type of ECMO	Timing of EN and PN nutrition		Complications	Mortality	Results	Nutrition tubes	Nutritional ingredients and requirements
Hofheinz et al. (3)	Prospective descriptive study; 15 were < 1 y of age	VA The median length of ECMO assistance was 10 d (6.5–13.5 d),	The first nutritional assessment was 2 d	The median time of PN was 2 d	EN: No complications were identified PN:Most were metabolic nature (hypernatremia, hypokalemia, hyperglycemia, and hypertriglyceridemia)	EN: 1/8 PN: 3/7, mixed PN/EN:4/7	EN appears to be safe in the setting of hemodynamic stability and absence of contraindications	High risk of aspiration:Post-pyloric feeding Low risk of aspiration:Gastric feeding	EN: infants <6 mo was breastfeeding or donor human milk, In older patients, polymeric formulas. Enteral tolerance: increasing the rate by 0,5 mL/Kg/h until the upper limit of fluid or caloric target
Hanekamp et al. (4)	Retrospective medical chart review; neonates (median gestational age was 40 wks)	VA The median duration of ECMO treatment was 7 (IQR, 5–9) days.	Hemodynamically stable situation and without stomach retention.	24 h after initiation of ECMO	EN was discontinued in 16/67 patients, with 14 showing gastric retentions, one showing discomfort, and one showing aspiration.	6of the 67 patients died	Neonates on ECMO tolerated enteral feeding well and did not show serious adverse effects.	Nasogastric feeding tubes were used in 58 patients (87%). 9 patients received EN through a transpyloric feeding tube.	EN: Breast milk was the primary source of food in all children. If not available, a regular infant formula was given. On the first day of EN, feeds were given as an hourly bolus of 1–4 mL, in 50% of the cases. The amount was daily increased PN: a combination of glucose 10% with 10% amino acids and lipids 20% (Intralipid 20%). Half the amounts of amino acids and lipids was given on the first day, 12 mL/kg/24 h and 6 mL/kg/24 h, respectively. On the second day, glucose with amino acids was given at 24 mL/kg/24 h and lipids 20% at 12 mL/kg/24 h.
Greathouse et al. (5)	A single center retrospective chart review; mean age 53 ± 76 months (range, 1 month–20 years)	VV was used in 11 (22%) cases	d5:any EN in 22 (45%) patients.	d5:PN was used in 27 (55%) of patients	EN: 1 patient having a bowel wall bleed on ECMO day 34. PN: Serious abdominal complications occurred in 5 of 49 patients (10.2%), 2 patients requiring drains for intraperitoneal hemorrhages, 1 patient requiring an exploratory laparotomy, and 1 abdominal compartment syndrome due to ascites from liver failure	Mortality during ECMO run PN:11 (41), EN:4 (18) In-hospital mortality,PN:16 (60) EN:6 (27; *p* = 0.025)	Initiation of EN by d5 was associated with improved survival to hospital discharge. Pediatric patients who received nutrition that was closer to goal energy intake, as well as those who received any EN early during ECMO, had improved survival to hospital discharge.		Due to fear of excess fluid, calorie-dense, high-protein formulas are often preferred over isotonic formulations. For term infants, protein needs were estimated using 2–3 g protein/kg/d. For pediatric patients, Protein needs for these patients are typically estimated at 2–4 g/kg/d.
Armstrong et al. (5)	Retrospective study Median (IQR) age was 0.1 (0, 16.4) months	VA, median duration was 8.5 (5.8, 24.3) days. venoarterial mode of ECMO were associated with lower EN delivery.	initiated by day 6 (2, 16). (35% postpyloric),	PN was initiated by day 1 (1, 3).	EN:GI intolerance was experienced by 53% (26 of 49); There were no suspected or confirmed necrotizing enterocolitis, gastrointestinal (GI) bleeding, or other major GI complication in the study population.	EN vs. no EN: 28-day mortality: 18.8% vs. 13.0%, 90-day mortality: 37.5% vs. 43.5%	Early EN is feasible in low volumes, but PN may be essential to prevent cumulative energy and protein deficits during the first week of ECMO.	32 patients(65%) had feeds delivered into the stomach, and the remaining 17 received postpyloric EN.	For neonates, a goal of 90–100 mL/kg/d was used with protein goal of2.5–3 g/kg/d. EN: 22 (67%)of these infants received EN in the form of breast milk, the remaining received formula PN composition was generally 55% carbohydrate, 20% protein, and 25% fat. Astandard pediatric parenteral multivitamin was provided daily to all patients
Pérez et al. (6)	Retrospective study; median age of 9.7 months (interquartile range [IQR] 3.9–63.1)	VA in 98% of the cases, the median duration of ECMO was 137.8hours (IQR 73.9–226.1hours)	Mean time from ECMO onset to the initiation of EN was 46.5 ± 40hours	Time to nutrition initiation (h)26.7 ± 24	EN developed digestive complications, they were mostly mild. Only three patients developed intestinal ischemia (one without EN and two on EN).	Mortality rate in the PICU: EN 37.7% PN:51%	EN is not associated with severe gastrointestinal complications or higher mortality. most prevalent complication was constipation in 31 patients (33%). EN, may be safe and beneficial in this population.	Transpyloric tube was the preferred method for EN delivery in these patients (97%) followed by continuous nasogastric tube (3% of the children).	Energy targets were calculated using Schofield equations for basal metabolic rate and protein targets were taken as the lower range of requirement for age in critically ill children. The formulas used were Isosource Junior in 25% of the patients, standard artificial formula in 20.3%, hypercaloric formulas in 14% and breast milk administered by feeding tube in 10.9% of the patients.
Alexander et al. (7)	Retrospective records; 76 children with median age (interquartile range [IQR]) of 0.3 years (0–2.6)	VA for ≥48 h	Fifty-five children (72%) started on EN by ECMO day 4 (IQR 3–7).	Median time (IQR) to starting PN was 2 days (1–3).	EN: Feeding intolerance was common, occurring in 38/55 (69%). Vomiting was the most common symptom of intolerance, occurring in 25/38 (66%).	Children able to achieve enteral autonomy were more likely to survive (*p* = 0.0024).	Initiation of EN by day 3 of ECMO and at a lower VIS(vasoactive-inotropic score.) is associated with greater likelihood of survival.		
Anton-Martin et al. (8)	Retrospective cohort study; neonatal and pediatric patients (N = 491) median age of 31 days (interquartile range, 2–771)	VA (88.2%); Duration of ECMO was a median of 154 h	median time from cannulation to enteral feeds initiation of 3 days (IQR, 1–4).	The median time from cannulation to TPN initiation was 1 day (IQR, 0–2)	EN were stopped in 26 (17.4%) of the 149 patients due to concern for pneumatosis intestinal is(occurred in 7.4% of patients)	Underweight (z score < −2): 65.8% Normal weight (−2 ≥ z scores≤ + 2): 48% Obese (z score > +2):45.4%	Underweight status was an independent predictor for in-hospital mortality in pediatric ECMO patients.		
Ohman et al. (9)	Retrospective study of neonates and children; the median age was 12 d [3 d, 16.4 y]	VA (70%) VV(30%)	Most patients received both enteral and parenteral nutrition at the time of ECLS initiation		Gastrointestinal complications occurred in 19.7% of patients including hemorrhage (4.2%), enterocolitis (2.5%), intra-abdominal hypertension or compartment syndrome (0.7%), and perforation (0.4%).	Mortality was 41%.	No relationship between route of nutrition and gastrointestinal complications (gastric, P ¼ 0.63; postgastric, P ¼ 0.34)		
Pettignano et al. (10)	Retrospective chart review;	7 supported with VA, All other 20 patients were supported withVV.	13 EN were initiated between 2 to 122 h (median 12 h) after the initiation of ECMO.	The remaining patients were started on PN between 12 and 48 h of the initiation of ECMO.	EN:All patients receiving EN had feedings interrupted: Distention 2 Increased residual 1 Cardiac arrest 1 Tube occluded 1 Sepsis in PN 2/14 and EN 1/13	EN 0/13 vs. PN 3/14	Enteral nutrition in patients receiving either venoarterial or venovenous ECMO is well tolerated		
Wertheim et al. (11)	Retrospective study; 96 neonates were treated with ECMO	VA for median duration of 161 h in EN group and 111 h in PN group	EN were introduced 30 to 138 h (median, 67 h) after the initiation of ECMO.		Gastric retention was observed in 4/16 patients in EN. EN and PN: systemic inflammatory response syndrome, 13% versus 6%; bacteremia, 6% versus 14%; sepsis, 6% versus 11%.	EN 0% *VS* PN 14%	EN is well tolerated and not associated with adverse effects.		Breast milk or standard formula through a nasogastric tube. Feedings are initiated at a rate of 0.5 to 1 mL/kg/h and increased each day by 0.5 to 1 mL/kg/h as tolerated.

## 3 Nutritional status and evaluation of ECMO children

### 3.1 Nutritional status

Children with ECMO have a higher risk of malnutrition (12), which is particularly prominent in children hospitalized in PICU. The study showed that nearly one-third of critically ill children had decreased nutritional indicators during their PICU stay. Retrospective studies have shown that the incidence of malnutrition in ECMO children is 33.3%, and the majority of them are under 2 years old (6). Anton-Martin (8) observed that 24% of 491 children with ECMO had a low body weight at the start of ECMO, low body weight was an independent risk factor for ECMO, and these children had a high in-hospital mortality. The stress response leads to muscle breakdown and amino acid release, exacerbating the problem of low body weight (13). Compared with adults, children have higher nutritional risks due to less nutritional energy reserves (14). The nutritional status of critically ill children is unstable and easy to deteriorate (15). pediatric ECMO patients show a trend of increased metabolism. Increased energy expenditure may lead to deterioration of the condition, resulting in prolonged mechanical ventilation and hospital stay. In addition, *in vitro* ECMO models have shown that the tubing itself may trigger pro-inflammatory and oxidative stress responses, increasing the risk of malnutrition in children (16).

Adequate feeding is typically defined as achieving 80–110% of the target value. The adequacy of nutrition provided for critically ill children during ECMO support is insufficient, particularly with regard to enteral nutrition (EN). EN may reduce gastrointestinal complications, but the proportion of calories and protein obtained through EN is lower, and the lower calories and protein from EN is because of the limited amount tolerated or provided (9). Additionally, a higher severity of illness score upon admission was found to be correlated with inadequate protein delivery (*p* = 0.040) (17). Study have shown (18) that when children have adequate energy intake (80% or more of predicted energy) and adequate protein intake (daily protein intake greater than or equal to 1.5 g/kg), their clinical outcomes are improved and the survival rate is higher. Moreover, protein adequacy may have a greater impact on children than energy intake, for example, more than 60% of predicted protein intake is associated with lower mortality in children with mechanical ventilation (19). In a multi-center study (20), the mortality rate was 9.3% in children receiving less than 20% of the predicted protein supply, 5.6% in those receiving 20 to 60, and 3.2% in those receiving more than 60%, indicating a positive correlation between survival and protein supply. Micronutrient and amino acid losses are marked during RRT in patients with AKI (21), To prevent a negative protein balance, enteral protein intake should be at least 1.5 g/kg/day, but currently there is insufficient evidence to support the need for additional supplementation of protein or amino acid intake during the acute phase. The nutritional status of ECMO children is closely related to clinical outcomes. Therefore, it is necessary to further study the nutritional adequacy of ECMO children and optimize the enteral nutrition strategy.

### 3.2 Evaluation indicators and tools

Various nutrition assessment parameters have been used to determine the presence and degree of malnutrition, including use of anthropometric measurements and laboratory values. Lipids are molecules involved in metabolism and inflammation. The plasma lipidome in critically ill children is very important to evaluate the nutritional status (22). Now, there are few studies on the relationship between lipids and nutrition metabolism in children with ECMO, but this provides a new perspective for further in-depth exploration. Carnitine is a key small molecule in the process of fatty acid oxidation and gluconeogenesis, and its deficiency can lead to significant complications in critically ill children. Study have shown (23) that the incidence of carnitine deficiency in ECMO children is higher than that in other hospitalized children, especially in children aged 1 week to 1 month. The level of carnitine is greatly influenced by nutritional support. About 75% of *in vivo* carnitine comes from dietary protein. The remaining carnitine can be synthesized endogenously in the liver, so older children are less prone to carnitine deficiency compared with neonates or premature infants (24). Evaluation of free carnitine levels may be appropriate for nutrition management, particularly if there are clinical concerns, such as unexpected hypoglycemia, cardiomyopathy, or chronic PN/dialysis use. Free carnitine <10 μM may reflect chronic deficiency and justify supplementation (25).

Currently, there is a lack of clarity on how to monitor and supplement carnitine, which points the way for future research. Thiamine is an essential coenzyme for aerobic glycolysis. Malnutrition may lead to thiamine deficiency, which in turn affects the symptoms of heart failure in children. Thiamine supplementation can significantly improve these symptoms (26). Thiamine supplementation is particularly important for high-risk children, especially those with malnutrition. In addition, in the case of thiamine deficiency, pyruvate is degraded by anaerobic metabolism, resulting in lactate formation and accumulation. Thiamine should be considered for treatment of children with unexplained lactic acidosis who are at risk for nutritional deficiencies. In hemodynamically stable children with elevated lactate, lactate levels can return to normal within 12 h after thiamine supplementation (27), allowing withdrawal of ECMO after 18 days. During ECMO treatment, more studies are needed to validate methods for blood lipids, carnitine, and thiamine measurements and the effects of their supplementation in critically ill children on the outcome deserve further investigations.

Body weight and basic nutritional status at admission are important for pediatric ECMO patients. Information on a child’s weight can be obtained by reviewing electronic medical records. Nutritional status should be evaluated within 24–48 h, which is not only helpful to monitor the changes in nutritional status of children, but also to assess the risk of nutritional deficiencies (28). The European Society for Pediatric and Neonatal Intensive Care (ESPNIC) recommends that the nutritional status of infants should be assessed regularly from the time of admission to the entire hospital stay (29). American Society for Parenteral and Enteral Nutrition (ASPEN) recommend that patients in the PICU undergo detailed nutritional assessment within 48 h of admission and that the nutritional status of patients be re-evaluated at least weekly throughout hospitalization (12). There are a variety of screening tools for nutritional status (such as the screening tool for risk on nutritional status and growth kids, STRONGkids) and screening tool for the assessment of malnutrition in pediatrics (STAMP). In the assesses, weight, height/length, mid-upper arm circumference and head circumference should also be included, and Z-score should be used to quantify it (29). The American Society for Parenteral and Enteral Nutrition (ASPEN) has developed an approach called nutrition-focused physical examination (NFPE), which focuses on nutrition and aims to detect fat and muscle loss early, thus reducing the severity of malnutrition. NFPE uses a head-to-toe approach to evaluate muscle mass, fat stores, fluid retention, micronutrient deficiencies, and functional capacity. However, its operation is relatively complex. A prospective cohort study (30) revealed that children who had lower nutritional complexity were more likely to complete the NFPE examination compared to those with higher nutritional risk. Moreover, older children were also more likely to complete the examination. Low complexity referred to patients needing basic nutrition education only, while high complexity referred to patients with chronic/complex diseases needing frequent reassessments or new enteral or parenteral nutrition interventions. The main challenges faced in completing NFPE were patient refusal and limited patient mobility/range of motion. Therefore, how to help children who need more time to complete NFPE due to mobility problems, nutritional complexity, and health related challenges and obtain their cooperation is the topic of future research.

## 4 Nutritional support during ECMO

### 4.1 Enteral nutrition

EN has significant benefits for maintaining gastrointestinal structure and functions. These include integrity and peristalsis function of gastrointestinal, intestinal immunity and absorption function, which helps to reduce the risk of liver injury and sepsis-related complications. A number of studies have shown that children using EN as initial artificial nutrition support during ECMO has a lower incidence of complications and a higher survival rate (28, 29, 31). For children with ECMO, early initiation of EN (within 48 h after ECMO initiation or when clinically stable) is safe (3). Regarding the complications of EN, most studies have shown that gastrointestinal complications are relatively mild. A 5-year retrospective study reported 77 children treated with ECMO, 67 of whom were fed successfully without bilious vomiting, bloody stools, or abdominal distention (4). Another study (32) showed that EN did not increase the risk of infection or abdominal complications in 49 ECMO children, and the closer the nutritional intake was to the target value, the better the survival rate of children. It is important to note that the duration of ECMO support and low cardiac output are related factors for death in ECMO children. High VIS (vascular activity-muscle strength score) is associated with increased mortality in children (33). However, some studies have come to different conclusions, it has been suggested that early EN may be beneficial for ECMO children, but there does not appear to be a clear association between nutrient and protein adequacy and clinical outcomes. Most studies believe that early EN administration is beneficial to ECMO children and is well tolerated (34, 35).

There is no clear definition of the timing of early EN. Some studies have suggested (36) that EN performed within 24–72 h after ICU admission is early stage. Buckvold et al. (37) pointed out that it is safe to carry out EN within 24 to 36 h. ESPNIC recommends that EN should be performed as early as possible for full-term neonates, critically ill children and children after cardiac surgery who are hemodynamically stable with extracorporeal life support (ECLS) or dependent on drugs. In principle, EN should be started within 24 h after admission and gradually increased until the nutritional goal is reached. The American Society of Critical Care Medicine and ASPEN define early enteral nutrition as 6 to 48 h after admission. To sum up, it is recommended to start EN within 48 h of ECMO support or as soon as the patient’s condition is stable. A reasonable goal (18) is to achieve 2/3 of the EN nutritional target within the first week, and to avoid intolerance, EN is usually started at a sustained low volume rate (i.e., 10-20 mL /kg/d) and increased slowly (i.e., every 6–12 h). If intolerance occurs, such as aggravated abdominal distension, obvious gastric retention, gastrointestinal gas, effusion, etc., enteral feeding should be stopped immediately and replaced by parenteral nutrition.

### 4.2 Parenteral nutrition

The generally accepted nutritional mode is early EN combined with supplemental parenteral nutrition. Armstrong et al. (5) adopted a strategy of parenteral nutrition combined with early low-dose enteral nutrition for ECMO children. Early parenteral nutrition combined with low-dose enteral nutrition is beneficial to achieve nutritional goals and reduce mortality of children. Jimenez et al. (38) showed that the reasonable nutrition method for ECMO children is early and slow EN as the choice of nutritional support. If the goal of nutritional delivery cannot be achieved by EN alone, it is recommended that children with malnutrition or low birth weight should receive PN support within 3 to 5 days. In order to prevent energy and protein deficiency during the first week of ECMO, parenteral nutrition support was initiated within 5 to 7 days in well-nourished Pediatric patients. For children with hemodynamic instability, ESPNIC recommends stopping parenteral nutrition within the first week (39). For children who need to use parenteral nutrition for a long time, to avoid catheter-related infection, it is recommended to use a separate PN venous catheter for infusion (40). However, a study revealed that no significant difference was found in the proportion of patients who acquired a new infection while on ECMO support between those receiving any EN vs. PN alone. More studies are needed to investigate the relationship between parenteral nutrition and the risk of infection in children. In an *in vitro* experiment (41), lipid emulsions were laminated and coagulated in the circuit 30 min after infusion, 73% ECMO centers preferentially used separate venous channels for lipid infusion, and 18% ECMO centers used ECMO circuit infusion, which resulted in agglutination, membrane dysfunction, and thrombosis. At present, most ECMO centers set the initial lipid requirement at 0.5 g/ (kg/d), which can be gradually increased to 2–3 g/ (kg/d) under the premise of monitoring triglyceride. In the early years of ECMO, the use of lipids was associated with circuit complications (i.e., circuit clotting, lipid deposition, and oxygenator dysfunction and failure). With the development of technology, the change of oxygenator membranes from microporous polypropylene membranes to “true” nonporous, polymethylpentene membranes, more recent experience suggests that lipid infusions are not associated with oxygenator failure (28).

### 4.3 Micronutrients

ECMO may lead to micronutrient deficiency. In the *in vitro* ECMO model, the essential amino acids isoleucine, vitamin A and vitamin E are lost from the pipeline (42). In animal models of acute lung injury induced by smoking, ECMO application is associated with selenium loss (43). The impact of these micronutrient deficiencies on ECMO treatment in children is important and requires further investigation. In children receiving ECMO and requiring dialysis, the loss of micronutrients is further aggravated, and continuous renal replacement therapy is associated with the loss of trace elements such as ionized calcium, inorganic phosphorus and selenium (44, 45). Calcium abnormalities frequently occurred in pediatric and neonatal patients undergoing ECMO support, and were found to be associated with a longer duration of ECMO and a longer length of ICU stay compared to patients who maintained normal calcium levels throughout ECMO therapy, with the underlying mechanism believed to be related to ECMO-induced disruption of normal calcium homeostasis (46). A 2014 American Society for Parenteral and Enteral Nutrition statement recommend an elemental calcium intake of 76 mg/kg and suggest a Ca:P ratio of 1.7:1 (mg:mg) or 1.3:1 (mmol:mmol) per day for short-term PN in neonates (47). Micronutrient loss in children with ECMO is an important area for future research.

### 4.4 The impact of CRRT

CRRT can restore euvolemia in neonates receiving ECLS and improve outcomes without increasing long-term renal morbidity (48). The primary indication for renal replacement therapy (RRT) during ECMO therapy is for either active volume management or fluid overload prevention in nearly 60% of cases (49). CRRT is beneficial for children with ECMO and may improve their nutritional status. Enteral nutrition is well tolerated in children treated with CRRT (50). During the first 72 h of ECMO, children receiving early CRRT consumed a large amount of protein with no significant change in glucose infusion rate, fat emulsion, or total energy (48). Children with ECMO are at risk for fluid overload (FO), and fluid removal or FO prevention may improve survival (51). In a multicenter cohort study of ECMO in children, FO was very common, and FO was associated with prolonged ECMO support and increased mortality (52). The above suggests that intervening before FO occurs may be a potential clinical therapeutic target. In pediatric literature, fluid overload has been associated with mortality and prolonged ECMO duration, while a negative fluid balance leads to improved respiratory function and time to weaning ECMO. CRRT can be used to treat AKI to alleviate fluid overload, which should be considered to improve outcomes in cases of patients with unsuccessful ECMO treatment (53). However, the literature on the nutrition required for ECMO combined with CRRT therapy is limited. CRRT depletes certain intravenous nutrients, and lead to macro- and micronutrient loss, especially protein loss (54, 55). Other CRRT-mediated losses may occur, including the loss of trace minerals, key nutrients, and water-soluble vitamins. Low serum albumin (ALB) is associated with the prognosis of severe AKI patients receiving continuous renal replacement therapy. The higher the serum ALB before CRRT, the lower the mortality of critically ill patients with AKI and treated with CRRT, and the higher the clearance efficiency of serum phosphorus (56). So, increased protein provision is likely necessary for neonatal ECLS patients if CRRT is utilized. However, no such guidelines exist for neonates. Further studies are necessary so optimal outcomes can be achieved for these patients.

## 5 Energy and protein requirements and calculations

Energy requirements for pediatric ECMO patients are similar to those of other critically ill children and can be estimated using the same methods as for other critically ill Pediatric patients (31). During the acute phase, energy intake should not exceed resting energy expenditure. After the acute phase, energy intake should take into account basic energy needs, physical activity, rehabilitation, and growth. Methods for calculating energy include isotope tracing, indirect calorimetry (IC), and Schofield formula. IC is internationally recognized as the gold standard for measuring energy expenditure but is not widely used due to its high monitoring costs and instability factors (57, 58).

Indirect calorimetry involves a pipette added to the patient’s respiratory system to measure expired CO2 and oxygen consumption. However, in ECMO patients, the oxygenator membrane removes CO2, rendering indirect calorimetry measurements unreliable.

Both American Guidelines for Nutritional Support Therapy in Critically Ill Children and China’s Guidelines for Nutritional Assessment and Support Therapy in Critically Ill Children recommend using the Schofield equation to estimate resting energy expenditure when IC measurement is not feasible.

The Guidelines for Nutritional Assessment and Supportive Treatment of Critically Ill Children recommend a minimum protein intake of 1.5 g/kg/d. The protein supply of ECMO children should be at least 1.5 g/kg/ day or higher intakes to prevent negative protein balance (59), gradually increasing to 3 g/kg/ day depending on age and severity to meet the needs of the body. Children are in a high catabolic state during ECMO and up to 3 weeks after ECMO support, and it is critical to compensate for protein loss by providing adequate protein, research has found (60) that protein breakdown is 100% higher in ECMO newborns than in healthy newborns of the same age and needs to be provided. It takes 1.5 g/kg/d of protein to achieve a positive nitrogen balance. While ASPEN recommends receiving a neonatal protein supply of 3 g/Kg/ day from ECMO to offset catabolic losses, ESPNIC believes that there is currently insufficient evidence that intake of 1.5 g/kg/ day or higher protein/amino acids during the acute phase of disease is beneficial for clinical outcomes. Accurate assessment of energy and protein requirements in children with ECMO is also an important area for future research. Based on previous studies, the nutritional assessment procedures for ECMO children were as follows: Nutritional assessment was performed within 24 h, energy requirement was calculated using Schofield formula, protein was at least 1.5 g/Kg/ day, and hemodynamic stability assessment was performed within 24 to 48 h, and the following conditions were assessed as stable: (1). The dosage of vasoactive drugs was stable; (2). Liquid resuscitation has been completed; (3). Stable blood lactic acid. Contraindications for EN should be further excluded in stable children: (1). Unrepaired congenital diaphragmatic hernia(CDH); (2). Severe intestinal obstruction; (3). Other abdominal contraindications. Pediatric patients with stable hemodynamics and no contraindications were given EN, otherwise PN was considered. EN implementation generally starts at 10 m1/Kg/ day, every 6 to 12 h, and reaches 80% of the required energy within a week. If the EN target does not reach 50% on the third to fifth day, nutrition is increased through PN.

## 6 Conclusion

Current research suggests that nutritional support for children with ECMO should follow the critical illness nutrition guidelines and take into account the specific needs. Early administration of enteral nutrition (EN) is a relatively safe practice, but due to the limited number of studies, further prospective, randomized controlled studies are needed to establish the best nutritional practices. PN (PN) plays an important auxiliary role. There has been some research progress, but no consensus has been reached. The micronutrient needs should also be of concern to children treated with ECMO. Continuous renal replacement therapy (CRRT) cannot be ignored in the nutritional support of ECMO children, and the rational application of CRRT is conducive to the nutritional management of children. More studies are needed to provide higher quality evidence to guide nutritional support.

## Author contributions

HZ: Conceptualization, Data Curation, Formal Analysis, Investigation, Writing – original draft, Writing – review & editing. LZ: Conceptualization, Data Curation, Formal Analysis, Investigation, Writing – review & editing. BJ: Data Curation, Formal Analysis, Investigation, Writing – review & editing.
